# Immunological Characteristics between αβ T _DC_ and γδ T _DC_ Cells in the Spleen of Breast Cancer-Induced Mice

**DOI:** 10.1055/s-0041-1730286

**Published:** 2021-06-02

**Authors:** Polyana Barbosa Silva, Márcia Antoniazi Michelin, Millena Prata Jammal, Eddie Fernando Cândido Murta

**Affiliations:** 1 Reseach Institute of Oncology, Universidade Federal do Triângulo Mineiro, Uberaba, MG, Brazil; 2 Discipline of Immunology, Universidade Federal do Triângulo Mineiro, Uberaba, MG, Brazil; 3 Department of Gynecology and Obstetrics, Universidade Federal do Triângulo Mineiro, Uberaba, MG, Brazil

**Keywords:** immunology, breast neoplasms, immunotherapy, T-lymphocytes, receptors, imunologia, neoplasias mamárias, imunoterapia, linfócitos T, receptores

## Abstract

**Objective** To evaluate the antitumoral role of γδ T _DC_ cells and αβ T _DC_ cells in an experimental model of breast cancer.

**Methods** Thirty female Balb/c mice were divided into 2 groups: control group ( *n* = 15) and induced-4T1 group ( *n* = 15), in which the mice received 2 × 10 ^5^ 4T1 mammary tumor cell line. Following the 28-day experimental period, immune cells were collected from the spleen and analyzed by flow cytometry for comparison of αβ T _DC_ (TCRαβ ^+^ CD11c ^+^ MHCII ^+^ ) and γδ T _DC_ (TCRγδ ^+^ CD11c ^+^ MHCII ^+^ ) cells regarding surface markers (CD4 ^+^ and C8 ^+^ ) and cytokines (IFN-γ, TNF-α, IL-12 and IL-17).

**Results** A total of 26.53% of γδ T _DC_ - control group ( *p* < 0.0001) - the proportion of αβ T _DC_ was lower in splenic cells than γδ T _DC_ ; however, these 2 cell types were reduced in tumor conditions ( *p* < 0.0001), and the proportion of IFN-γ, TNF-α, IL-12 and IL-17 cytokines produced by γδ T _DC_ was higher than those produced by αβ T _DC_ , but it decreased under conditions of tumor-related immune system response ( *p* < 0.0001).

**Conclusion** Healthy mice engrafted with malignant cells 4T1 breast tumor presented T _DC_ with γδ TCR repertoire. These cells express cytotoxic molecules of lymphocytes T, producing anti-tumor proinflammatory cytokines.

## Resumo

**Objetivo** Esclarecer o possível papel antitumoral das células T _DC_ γδ e T _DC_ αβ em um modelo experimental de câncer de mama.

**Métodos** Trinta baços de camundongos Balb/c analisados por citometria de fluxo, separados entre grupo controle ( *n* = 15) e o grupo tumoral induzido por 4T1 ( *n* = 15).

**Resultados** Presença de 26,53% de T _DC_ γδ nos camundongos do grupo controle ( *p* < 0,0001), proporção de T _DC_ αβ menor em células esplênicas do que T _DC_ γδ; no entanto, estes dois tipos de células são reduzidos em condições tumorais ( *p* < 0,0001), e a proporção de citocinas IFN-γ, TNF-α, IL-12 e IL-17 produzidas pelas célula T _DC_ γδ foi maior do que as produzidas pelas células T _DC_ αβ, mas foram diminuídas sob condições de resposta ao sistema imunológico relacionada ao tumor ( *p* < 0,0001).

**Conclusão** Camundongos saudáveis induzidos ao tumor de mama 4T1 apresentaram T _DC_ com repertório TCR γδ. Estas células expressam moléculas citotóxicas de linfócitos T, produzindo citocinas proinflamatórias anti-tumor.

## Introduction

A new type of immune cell has been described, and these new cells have characteristics of innate and acquired immunity. T _DC_ cells were identified in mice and in humans as cells that express T cell receptors (TCRαβ) specific of T lymphocytes, and simultaneously express the CD11c markers and major histocompatibility complex class II (MHCII or HLA [Human Leukocyte Antigen] in humans), found in innate cells, mainly in dendritic cells. These molecules in the same cell confer unique characteristics and properties; they carry out functions of dendritic cells (DCs) that do not need to be activated by antigen-presenting cells (APCs). When stimulated by specific receptors, such as the family of Toll-like receptors, they can produce interleukin-2 (IL-12) cytokine, as well as process and present antigens. 1

T lymphocytes respond in a specific manner to pathogens and cancer cells by the recognition of specific antigens due to TCR (T-cell receptor) in their membrane, similar to the role of the immunoglobulins in B cells. The TCR consists of 2 polypeptide chains; ∼ between 90 and 99% of all T cells have the αβ TCR, but a minority has γδ chains. 2 3 Both cells originate from common thymic precursors, but the biological roles and molecular understanding of these two subsets differ substantially. The T lymphocytes that express αβ TCR depend on the presentation of antigens in a defined HLA molecule to be activated, and usually are tolerant to self-peptides. On the other hand, the γδ T lymphocytes do not rely on the recognition of classic HLA molecules, and the identification of tumor antigen is made by ubiquitous changes observed across many individuals, which allows these cells to not undergo the rejection process, and, consequently, they can be transferred more easily between individuals. Unlike αβ T cells that have their biological role well-characterized in cancer immune surveillance, the protective role of γδ cells during tumor development has only been increasingly reported over the past two decades.

The presence of tumor-infiltrating γδ T lymphocytes has been associated with good prognosis in patients with melanoma 4 and gastric cancer, 5 and high levels of these types of circulating lymphocytes have been associated with reduced cancer risk, increased 5-year-disease-free and increased survival after bone marrow transplant for acute leukemia. 6

The antitumoral ability of γδ T lymphocytes is associated with their synthesis of interferon γ (IFN-γ), and of tumor necrosis factor-α (TNF-α), as well as their cytotoxic potential. Other studies have also reported the role of interleukin-17 (IL-17) produced by γδ T cells, mainly when they act together with immunogenic cell death-inducing chemotherapeutic drugs. 7

To clarify whether T _DC_ cells could also have the γδ chains and the possible antitumor role of this new cell population, we investigated the T _DC_ population comparing both αβ TCR and γδ TCR T _DC_ , as well as their cytokines in an experimental model of mice engrafted with malignant cells.

## Methods

### Animals

Thirty 8-week-old female Balb/c mice, kept in the sectoral vivarium of the Oncology Research Institute (IPON, in the Portuguese acronym) of the Universidade Federal do Triângulo Mineiro (UFTM, in the Portuguese acronym), were used. During the 28-day experimental period, the animals were divided into a control group (healthy mice) and a tumor group (4T1 breast tumor cell-engrafted mice). Each group consisted of 15 animals, was housed in plastic cages under a 12-hour light/dark cycle at 21 ± 3°C, with food and water available ad libitum. After the experimental period, the animals were euthanized by overdosing with 50 mg/kg of ketamine and 15 mg/kg of xylazine, and their spleens were removed for analysis. The present study was approved by the Ethics Committee on Animal Use of the UFTM, under number 379/2016 - CEUA/UFTM.

### Tumor

The animals were selected at random, and the tumor-induced group was engrafted with 4T1 inoculated with 2 × 10 ^5^ cells in the last pair of breasts, on the left mammary gland. Tumor cells of the strain mentioned above are cells isolated from the Balb/c mice spontaneous tumor, with high proliferative, invasive, and tumorigenic power. The cells were maintained in culture in Roswell Park Memorial Institute (RPMI) medium and incubated at 37°C and 5% CO 2 (Water Jacket Incubator 3110, Thermo Fisher Scientific, Marietta, OH, USA). After the culture period, the cells were washed with 0.9% saline solution and centrifuged at 290xg at 4°C for 10 minutes and then inoculated in the group of tumor-induced by 4T1 mice.

### Characterization of Immune Cells by Flow Cytometry

The spleens of the control group and of the tumor group were disclosed, filtered, and washed with saline solution, and after counting in a Neubauer chamber, 1 × 10 ^6^ cells were placed in tubes suitable for the flow cytometry technique. The cells were then labeled with extracellular anti- γδ TCR- antibodies (T lymphocyte receptor), anti-CD11c (adhesion molecules), anti-IA (antigen-presenting molecule), anti-CD4 (helper T lymphocytes), and anti-CD8 (cytotoxic T lymphocytes) – all antibodies acquired from BD Biosciences. After the 30-minute incubation, the cells were washed and prepared to receive the intracellular antibody labels for anti-IFN-γ, anti-TNF-α, IL-12, and IL-17 proinflammatory cytokines. To block nonspecific binding, the antimouse IgG2b Immunoglobulin G2b) - (mouse) Rabbit Monoclonal Antibody (IgG2b), anti-rat IgG2a Immunoglobulin G2a - (mouse) Rabbit Monoclonal Antibody (IgG2a), and antirat IgG2b isotypes were used. The cells were read on the BD FACSCalibur (BD Biosciences, Franklin Lakes, NJ, USA) cytometer, and the data analyzed using Flowing software.

The gating strategy used was the delimitation by size and granularity (FSCxSSC) of the spleen cells of the control and tumor groups. Subsequently, the double-positive labeling of CD11c and IA (MHCII [Major Histocompatibility Complex - class II]) was limited and, thus, the γδ TCR labeling traced the γδ T _DC_ cells. Within this population of γδ T _DC_ , we analyzed the phenotypic and cytokine markers of interest.

### Statistical Analysis

Statistical analyzes and graphs were prepared using GraphPad Prism 5.0 (GraphPad Software, San Diego, CA, USA). The Kolmogorov-Smirnov tests were used to verify the normality of the variables. Non-normal samples were analyzed by the Mann-Whitney test, both for comparison between the control group and the tumor-induced group from both profiles and for the comparison of αβ T _DC_ and γδ T _DC_ cell expression. The data obtained were represented with their corresponding median, minimum and maximum values. The difference found between the groups was considered statistically significant when *p* < 0.05.

## Results

**Fig. 1:**
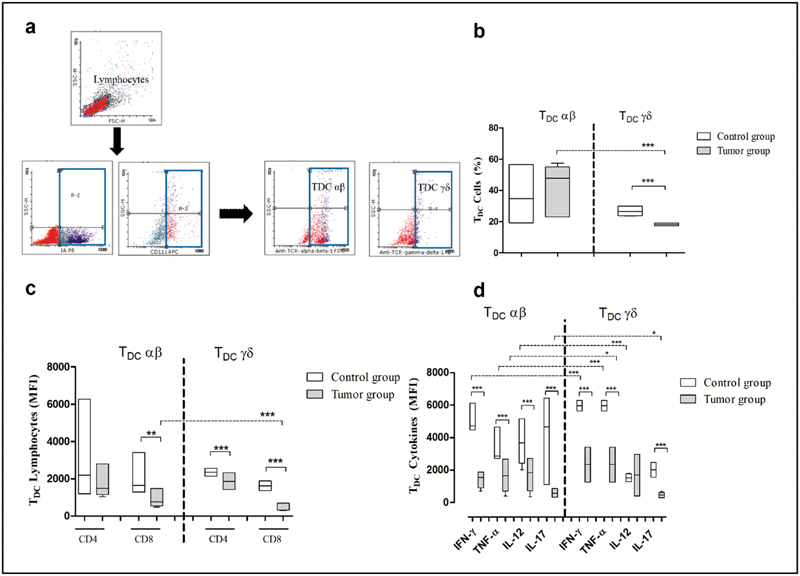
Comparison of immunological characteristics between αβ T _DC_ and γδ T _DC_ cells in the control and in the tumor group. (a) Representative graph of flow cytometric analysis to identify frequency of αβ T _DC_ and γδ T _DC_ cells in the spleen of the control and of the tumor group. (b) Frequency analysis of αβ T _DC_ and γδ T _DC_ cells in the spleen of the control and of the tumor group. (c) Mean fluorescence intensity of αβ T _DC_ CD4 ^+^ /γδ T _DC_ CD8 ^+^ and γδ T _DC_ CD4 ^+^ /γδ T _DC_ CD8 ^+^ cells in the spleen of the control mice and of the tumor group. (d) Mean fluorescence intensity of αβ T _DC_ IFN-γ, αβ T _DC_ TNF-α, αβ T _DC_ IL-12, and αβ T _DC_ IL-17 and γδ T _DC_ IFN-γ, γδ T _DC_ TNF-α, γδ T _DC_ IL-12 and γδ T _DC_ IL-17 cells in the spleen of the control and of the tumor group. Representative graphs of two independent experiments, *n* = 15 each (median with range). The results were analyzed by the Mann-Whitney test to compare the mean fluorescence intensity of subtypes αβ T _DC_ and γδ T _DC_ cells (statistical differences represented by the dashed line). Differences were considered statistically significant at *p* < 0.05 (5%). * *p* < 0.05; ** *p* < 0.001; *** *p* < 0.0001.

The flow cytometry profile shows the comparison of αβ T _DC_ (TCRαβ ^+^ CD11c ^+^ MHCII ^+^ ) and γδ T _DC_ (TCRγδ ^+^ CD11c ^+^ MHCII ^+^ ) cell infiltrates in the spleen of healthy mice engrafted by breast cancer 4T1 cells ( Figs. 1 a, b, c and d ). When analyzing the frequency of the γδ T _DC_ cell profile ( Fig. 1a ), a significant decrease was found in the tumor group, with a median of 18.11 (17.21–19.01) compared with the control group (26.53; 23.62–29.99) ( *p* < 0.0001). The frequencies of both αβ T _DC_ and γδ T _DC_ cells were compared, and a significance was found in the tumor-induced group of both cell profiles, that is, there was a higher amount of αβ T _DC_ cells (47.74; 22.97–57.36) than of γδ T _DC_ cells (18.11; 17.21–19.01) in the spleen of the 4T1 tumor-induced mice group ( *p* < 0.0001).

The mean fluorescence of auxiliary T lymphocyte (CD4) and cytotoxic T lymphocyte (CD8) markers present in the αβ T _DC_ and γδ T _DC_ cells of both groups ( Fig. 1b ) was analyzed, and it was observed that the CD8 αβ T _DC_ cells showed a decrease in the tumor group (764.7; 485.8–1467) in comparison with the control group (1,650; 1,292–3,418) ( *p* = 0.0012). Regarding CD4 γδ T _DC_ cells, a significant decrease was found in the tumor group (1,873; 1,421–2,325) compared with the control group (2,350; 2,140–2,561) ( *p* = 0.0009), as well as for CD8 γδ T _DC_ cells, of which there was significant decrease in the 4T1 tumor group (329.0; 292.3–692.4) compared with the control group (1,630; 1,370–1,889) ( *p* < 0.0001). When comparing both cell profiles, a decrease of CD8 γδ T _DC_ cells (329.0; 292.3–692.4) was found in comparison with CD8 αβ T _DC_ cells (764.7; 485.8–1467) ( *p* < 0001) in the tumor group.

The mean fluorescence intensity of the cytokines produced by αβ T _DC_ and γδ T _DC_ cells were analyzed ( Fig. 1c ). It was noticed that, for IFN-γ αβ T _DC_ cells, there was a decrease in the tumor group (1,554; 705.3–1,885) compared with the control group (4,720; 4,488–6,120) ( *p* < 0.0001), as well as a TNF-α αβ T _DC_ decrease in the tumor group (1,655; 403.8–2,673) compared with the control group (2,877; 2,716–4,658) ( *p* < 0.0001). Regarding IL-12 αβ T _DC_ cells, a decrease was observed in the tumor group (1,841; 360.7–2,728) compared with the control group (3,686; 2,028–5,163) ( *p* = 0.0002), as well as for L-17 αβ T _DC_ cells, which also decreased in the tumor group (578.5; 326.3–873.8) compared with the control group (4,666; 1,117–6,436) ( *p* < 0.0001).

In relation to the cytokines produced by γδ T _DC_ cells, a decrease in IFN-γ γδ T _DC_ cells was found in the tumor group (2,349; 1,261–3,429) compared with the control group (5,972; 5,649–6,297) ( *p* < 0.0001). Regarding IL-17 γδ T _DC_ cells, a decrease in the tumor group (468.3; 307.1–692.2) was observed when compared with the control group (2,026; 1,563–2,489) ( *p* < 0.0001).

Finally, the profiles of αβ T _DC_ and γδ T _DC_ cells were compared, and an increase in the IFN-γ γδ T _DC_ cells of the control group (5,972; 5,649–6,297) was found, when compared with IFN-γ αβ T _DC_ cells (4,720; 4,488–6,120) ( *p* = 0.0005). An increase was also found in the TNF-α γδ T _DC_ cells of the control group (5,972; 5,649–6,297) compared with TNF-α αβ T _DC_ cells (2,877; 2,716–4,658) ( *p* < 0.0001), as well as an increase in TNF-α γδ T _DC_ cells in the tumor group (2,349; 1,261–3,429) in relation to TNF-α αβ T _DC_ cells (1,655; 403.8–2,673) ( *p* = 0.0157). There was a decrease in IL-12 γδ T _DC_ cells in the control group (1,526; 1,290–1,793) compared with IL-12 γδ T _DC_ cells (3,686; 2,028–5,163) ( *p* < 0.0001). When comparing IL-17 γδ T _DC_ cells, a decrease was found in the tumor group (468.3; 307.1–692.2) in relation to IL-17 αβ T _DC_ cells (578.5; 326.3–873.8) ( *p* = 0.0157).

## Discussion

Kuka et al 1 described T _DC_ cells (TCRαβ ^+^ CD11c ^+^ MHCII ^+^ ) as a cell subtype with properties common to polyclonal T αβ cells and dendritic cells. These rare cells have a morphological similarity to dendritic cells that express intermediate levels of CD11c and present Major Histocompatibility Complex (MHC) class II antigenic molecules. Besides, these cells are also characterized by the expression of costimulatory molecules (CD80, CD86) and lymphocyte surface markers (CD3, CD4, and TCR α/β). 1

The frequency of αβ T _DC_ cells described by Kuka et al 1 is of ∼ 0.04% in the spleen of healthy mice. In our study, it was identified an average of 34.64% in healthy mice and of 47.74% in the group of 4T1 breast tumor cell-engrafted mice. Kuka et al 1 identified and characterized T _DC_ cells by flow cytometry by analyzing the total cells. Our study delimits an area (gate) referring to lymphocytes, size and granulation of this cell type. T _DC_ cells have similar morphology and size to T lymphocytes. 1

The presence of the cell profile for γδ T _DC_ (TCRγδ ^+^ CD11c ^+^ MHCII ^+^ ) was verified in the same conditions, and the presence of a percentage of 26.53% of γδ T _DC_ in the control group and of 18.11% in mice with breast cancer ( *p* < 0.0001) was found.

The present study reports that between 1 and 4% of all T cells present in the thymus, in secondary lymphoid organs, and in the lungs of adult mice are γδ T lymphocytes. In mucous membranes, such as the intestinal membrane, there are 25 to 40% of this cell type, where the most significant amount is concentrated, 8 in addition to presenting subtypes as well as phenotypic and functional dieting properties. 9

In our studies, the effect of a systemic immune response under the influence of tumor cells, which decreased both αβ T _DC_ and γδ T _DC_ cells, was observed. However, when comparing these two cell profiles – αβ T _DC_ and γδ T _DC_ – there was a higher amount of αβ T _DC_ in the 4T1 tumor-induced tumor group than γδ T _DC_ ( *p* < 0.0001).

The αβ T cell repertoire is higher in T lymphocytes and, most of the time, they have protective antitumor activity, mainly related to human melanoma tumors. 10

A study with human blood samples from 38 patients diagnosed with breast cancer compared with healthy controls showed that the proportion of γδ T cells in the circulating blood of healthy controls is 1.6 times greater than in breast cancer patients. 11 These data corroborate with our study, since γδ T _DC_ cells are present in more significant quantities in the control group ( *p* < 0.0001).

Concerning γδ T cells, in an antitumor immune response, pioneering studies on the immunoprotective role of these cells in mice were performed in murine models with skin cancer, which were chemically induced by carcinogens or by subcutaneous transfer of melanoma tumor lineage. From these studies, relevant roles of γδ T in antitumor immunity have been described, with mechanisms mediated by the NKG2D C-type lectin-like receptor expressed on NK (NKG2D) receptor by dendritic epidermal T cells (DETCs)Vγ5 + residing in tissues. 12 13

Studies comparing tumor progression in mice with deficient γδ T cells (due to genetic inactivation of the γδ TCR receptor) versus mice with sufficient γδ T cells (wild) have firmly established the protective role of γδ T cells 10 because it was found that γδ T cells prevented the progression of chemically induced papilloma to cutaneous squamous cell carcinomas. In contrast, αβ cells seemed to favor tumor progression 14 ; the same happened with spontaneous B cell lymphomas, 15 prostate cancer 16 and in the transplantable model of melanoma B16-F0. 17 Besides, some studies show, in the context of infections by cytomegalovirus and malaria, that γδ T cells can be activated later, in the form of direct cytotoxicity, by the action of granzyme B and through stimulating effects such as the secretion of cytokines IFN-γ and TNF-α, or by the direct presentation of antigen. 18

Most γδ T cells, unlike αβ T lymphocytes, do not exhibit CD4 or CD8 coreceptors, so antigen recognition is not restricted to antigen-presenting molecules. 8 Thus, the expression of αβ T _DC_ and γδ T _DC_ cells related to helper T lymphocyte (TCD4) and cytotoxic (TCD8) markers was compared, revealing that the proportion of CD8 γδ T _DC_ is less expressed in splenic cells than CD8 αβ T _DC_ , but these two cell types are decreased in tumor conditions ( *p* < 0.0001).

A recently conducted study comparing subsets of γδ T lymphocytes in 40 patients with Chron disease demonstrated a significant decrease in this cell population, concluding that this condition can affect the immune responses against this disease. 19 We believe that, like cancer, the suppressive conditions provided by them can have the same result with αβ and γδ T _DC,_ leading to a deficiency of this mechanism.

The central cytokines produced by γδ T _DC_ presented a higher proportion of this cell type than those produced by αβ T _DC_ . That is, there is a higher production of the IFN-γ, TNF-α, IL-12 cytokines in the control group, and a lower proportion of IL-17 γδ T _DC_ in the group of mice with breast cancer. However, this condition decreases when there is a systemic immune response related to tumors ( *p* < 0.0001).

It is inferred that γδ T _DC_ cells are similar to the mechanisms exerted by γδ T cells. Studies show that this cell type is an important precursor source of IFN-γ and TNF-α, which inhibits tumor growth and angiogenesis. Also, the study performed with the combination of concanavalin A (ConA) and interleukin-2 (IL-2) demonstrated the potential for polarization and plasticity of γδ T _DC_ cells, which induced the intense proliferation of these cells and the consequent production of interleukin-12 (IL-12) and interleukin-2 (IL- 18). 20

In inflammatory conditions, a situation observed in some cancers and infections, they favor the polarization of γδ T cells toward an IL-17 producing phenotype. 21 A recent study of transcriptome sequencing in ∼ 18,000 tumor masses in humans revealed that, among tumor-infiltrating leukocytes, γδ T cells were strongly associated with a good prognosis. 22 In our study, it was observed that γδ T _DC_ in a systemic immune response is suppressed regarding αβ T _DC_ cells in tumor conditions ( *p* = 0.0157).

A specific type of γδ T cells (γδ TCD27 ^+^ ) from mice secrete the IFN-γ cytokine, responsible for inhibiting tumor angiogenesis and improving the expression of MHC class I by tumor cells, thus promoting efficiency in the responses of CD8+ T cells. 23 In our studies, it was observed that γδ T _DC_ cells expressed IFN-γ in more significant quantities in healthy mice ( *p* < 0.0001). In the study with a model of adoptive transfer of γδ T cells in mice against melanoma B16-F0, it was observed that a specific subtype of γδ T Vγ4 ^+^ (but not Vγ ^+^ T cells) had the protective function dependent on its high eomesodermin expression and IFN-γ production. 24

Even though IFN-γ is the main cytokine produced by mouse γδ T cells, IL-17 is involved in the protective responses of γδ T cells in some cancer models. 25 Interleukin-17-producing γδ T cells cooperated in mediating bladder cancer regression. 26 In another study, IL-17-producing γδ T cells are associated with chemotherapeutic agents (such as doxorubicin) in various models of epithelial tumor transplantation and demonstrated a better antitumor response. 27 In our study, it was identified that IL-17-producing γδ T _DC_ is less frequent in the breast cancer-induced group compared with IL17-producing T _DC_ αβ ( *p* < 0.0001). Thus, it can be inferred that, in a systemic antitumor response, these cells may be suppressed by tumor escape mechanisms to antitumor immune responses.

Therefore, according to the data found in the present study, we can conclude that γδ T _DC_ has immunological characteristics shared with conventional effector αβ T _DC_ cells. The healthy mice engrafted with 4T1 breast tumor presented T _DC_ with γδ TCR repertoire. These cells express T helper and cytotoxic T lymphocyte molecules, producing antitumor proinflammatory cytokines, suggesting that γδ T _DC_ could have an antitumor role, and even be used in the future in antitumor immunotherapy. However, new studies investigating its function in other tumor types are necessary.
